# Mass Rearing History and Irradiation Affect Mating Performance of the Male Fruit Fly, *Anastrepha obliqua*

**DOI:** 10.1673/031.012.4501

**Published:** 2012-03-29

**Authors:** Juan Rull, Nery Encarnación, Andrea Birke

**Affiliations:** ^1^ Instituto de Ecología, A.C., Apartado Postal 63, 91000 Xalapa, Veracruz, Mexico; ^2^ Benemérita Universidad Autónoma de Puebla, Puebla, Mexico

**Keywords:** sterile insect technique, competitiveness, Tephritidae

## Abstract

As an initial step to improve the efficiency of the sterile insect technique applied to eradicate, suppress, and control wild *Anastrepha obliqua* (Macquart) (Diptera: Tephritidae) in mango producing areas of Mexico, the effect of radiation dose and mass rearing history on male mating performance was examined. Field cage tests in which both male and female laboratory flies were irradiated at different doses (0, 40, and 80 Gy) were released with cohorts of wild flies of both sexes, revealing that both mass rearing history and irradiation affected male mating performance. Laboratory males were accepted for copulation by wild females less frequently than wild males. Copulations involving laboratory males were shorter than those involving wild males. Irradiated males mated less frequently with wild females than wild males, and irradiated females appeared to be less able to reject courting males of both origins. High levels of fertility for untreated laboratory females crossed with males irradiated at different doses may reflect problems in mass rearing affecting homogeneity of pupal age before irradiation, and possibly masked a dose effect. Proposed remedial measures to improve male mating performance are discussed.

## Introduction

The sterile insect technique (SIT) is a biologically based pest control method which involves the rearing, sterilization, and release of large quantities of insects into a target pest population to induce sterility (Knipling 1979). Although SIT is most often associated with eradication, more recent advances in rearing efficiency, handling, and release methods have made the use of sterile insects economically feasible for suppression, prevention, or containment (Vreysen and Robinson 2011). SIT has been applied to eradicate or control a wide variety of pest species, including screwworm *Cochliomyia hominivorax* (Wyss 2000), tse-tse fly *Glossina palpalis palpalis* (Oladunmade et al. 1990), codling moth *Cydia pomonella* (Bloem et al. 2005), pink boll worm *Pectinophora gossypiella* (Henneberry 2007), melon fly *Bactrocera cucrbitae* (Kuba et al. 1996), medfly *Ceratitis capitata* (Hendrichs et al. 2002), and Mexican fruit fly *Anastrepha ludens* (Gutiérrez-Ruelas and Santiago 2008), and its use is currently being expanded to include mosquitoes (Alphey et al. 2010).

In Mexico, SIT has been applied for fruit fly eradication and control since 1978 (Rull et al. 1996). Current efforts relying on SIT in conjunction with other techniques, such as innundative releases of parasitoids (Montoya et al. 2000), are in use to eradicate, suppress, and control the Mexican fruit fly (Gutiérrez-Ruelas and Santiago 2008) and have recently been expanded to include the West Indian fruit fly *Anastrepha obliqua* (Artiaga-Lopez et al. 2004).

The West Indian fruit fly, *A. obliqua*, is widespread in the Neotropics, occurring in Mexico, Central and South America, and the Caribbean islands The preferred host plants of this species are in the genus *Spondias* (hog plums) (Aluja and Birke 1993), but it is also the most important pest of mangos in Mexico and tropical America (Peña et al. 2009). Mexico is the largest exporter of mangoes in the world (Mukherjee and Litz 2009). Efforts to control *A. obliqua* through SIT and other area-wide methods in mango growing areas of Mexico have been applied for more than nine years (Hernández et al. 2009). From 2000–2008, 130,000 tons of mango were produced in fruit fly-free areas, low prevalence areas, and areas under phytosanitary control (Gutiérrez-Ruelas and Santiago 2008).

Despite success, recent findings have corroborated that mass rearing history and radiation dose can affect performance of sterile males of several fruit fly species used for SIT (Hendrichs et al. 2002; Lux et al. 2002; Rull et al. 2005; Collins et al. 2008, 2009). In the case of *A. ludens*, reduction of radiation doses and colony refreshment schemes (Rull et al. 2007; Rull and Barreda-Landa 2007) are viable measures to restore competitiveness of released males. In the case of *A. obliqua*, although irradiation has been found to affect sterility induction in the laboratory (Toledo et al. 2004), dispersal ability in the field (Hernández et al. 2007), and demographic changes caused by artificial selection (Hernández et al. 2009), no field cage experiments have been performed to examine their effect on sexual performance of irradiated mass reared males. This is particularly important considering ongoing efforts for population suppression using SIT in northwestern Mexico and the fact that the *A. obliqua* strain produced by the national fruit fly campaign is nine years old (Hernández et al. 2009). Furthermore, Toledo et al. (2004) suggested that in areas where native pests are well established (the case of *A. obliqua* in México) lowering radiation doses will result in greater sterility induction through increased male performance with little risk of spreading fertile males. Under an early stage suppression scenario, no risk is incurred as long as the reduced dose is sufficiently high to guarantee full female sterility; in the case of *A. obliqua*, no genetic sexing strain has been developed, and females are more radiosensitive than males (Toledo et al. 2004).

As an initial step to improve efficiency for *A. obliqua* management and eradication, this study investigated the effect of mass rearing history (strain) and radiation doses on laboratory male mating performance when competing with wild males for matings with wild and laboratory females.

## Materials and Methods

### Biological material

Wild *A. obliqua* were recovered from infested *Spondias mombin* L. (Sapindales: Anacardiaceae) collected in the locality of Llano Grande, Veracruz, during September 2010. Pupae were recovered from infested fruit following methods described in Aluja et al. (2003) and left in 200 mL plastic mesh covered cups lined with a thin layer of vermiculite under controlled environmental conditions of 27 °C, 65% RH, 12:12 L:D until adult emergence. At emergence, adult wild flies were sexed and placed in 30 × 30 × 30 cm Plexiglass cages with free access to water and food as described in Rull et al. (2007).

Laboratory flies were obtained from the Moscafrut mass rearing facility according to methods described in Artiaga-López et al. (2004). 48 hours before adult emergence, three lots of mass reared pupae were packed in plastic bags, sealed, and subjected to radiation. One lot remained untreated (0 Gy), a second lot received a dose of 40 Gy, and a third lot received the standard dose of 80 Gy. Radiation was applied using a Gammacel 200 irradiator (MDS Nordion, www.nordion.com). Mass reared pupae were shipped to Xalapa, and in all cases hypoxia was broken after 12 hours. Two days later, at the beginning of adult emergence, adults were sexed and placed in Plexiglass cages as described above. Adult emergence spanned 6 days, with a peak of emergence between days 3 and 4. Adults were left in cages in groups not exceeding 60 individuals until they reached sexual maturity (10–15 days in the case of laboratory flies and 15–20 days in the case of wild flies).

### Field cage tests

Two days prior to testing, wild and laboratory adults were marked on the back of the thorax with a small dot of acrylic paint according to origin and treatment, following methods in Rull et al. (2007). On test days, males were released early in the morning (06:30–07:00) in three 2 × 1 × 1 m field cages containing three potted citrus and mango trees. Half an hour later females were also released in cages and observations began. Each cage received 30 wild males, 30 wild females, 30 laboratory males, and 30 laboratory females. Laboratory flies were either untreated (0 Gy) or irradiated at 40 or 80 Gy.

In each cage an observer removed mated couples with a small plastic vial, recording male and female color and the time at which copulation began. Couples in vials were placed on the side of field cages and watched regularly to record end of copulations. The experiment was replicated seven times, rotating in each case the cage in which a particular radiation treatment was tested.

### Fly sterility testing

In order to evaluate the level of sterility of non-irradiated males, and males irradiated at 40 and 80 Gy, ten treated males and ten non-irradiated laboratory females were introduced in three 2 L plastic cages. A 2.5 cm agar sphere, as described in Díaz-Fleischer and Aluja (2003), was hung from the cage ceiling of each cage. Every 24 hours for three days, 30 eggs were removed from spheres and aligned over a dark piece of cloth placed over a moist cotton pad inside a 9 cm-diameter Petri dish. Because *A. obliqua* females lay a single egg per oviposition bout, and spheres typically received more than 30 eggs during the three-day period, the sampling method allowed for an accurate estimation of induced sterility. Eggs were incubated at 33 °C for five days and observed under a microscope to record the number of hatched eggs. Spheres were replaced each day for three days (until 90 eggs were observed). The experiment was replicated four times.

### Statistical analysis

Mating propensity was analyzed by building a general linear model (GLM) followed by a factorial ANOVA with log (x + 1) transformed counts (number of copulations per replicate) as the dependent variable and using treatment (0, 40, and 80 Gy) and mating combination (Wild ♂ × Wild ♀; Wild ♂ × Lab ♀; Lab ♂ × Wild ♀; Lab♂ × Lab ♀) as factors. The degree of mating isolation between wild and laboratory flies was estimated for each radiation dose by calculating ISI, MRPI, and FRPI isolation indices following Cayol et al. (1999) (see Table 1 footnote for details). Mating duration in minutes was compared among mating combinations for each treatment by building a linear model followed by a factorial ANOVA using treatment and mating combination as factors. Copulations lasting less than 10 min or more than two hours were removed from the analysis. Treated male sterility (egg hatch) was compared among treatments with a repeated measures ANOVA on arc-sin transformed proportions. All analyses were done using Statistica 7® (StatSoft Inc., www.statsoft.com).

## Results

### Field cage tests

**Total matings**. A factorial ANOVA revealed a significant effect of mating combination on number of copulations per replicate (F_3,72_ = 3.47, *p* < 0.05) and no significant effect of treatment or the interaction between mating combination and treatment (*F*_2,72_ = 0.75, *p* = 0.475), (*F*_6,72_ = 0,88, *p* = 0.508), respectively. Irrespective of treatment (0, 40, or 80 Gy), laboratory males mated less frequently with wild females when compared to other mating combinations. Mating isolation index values for different radiation doses are shown in Table 1. ISI values revealed a slight tendency towards assortative mating between non-irradiated and wild flies. In the case of irradiated flies (both doses) there was tendency for irradiated females to mate with greater frequency than wild females (FRPI values), but they did so with males of both origins.

**Table 1.**
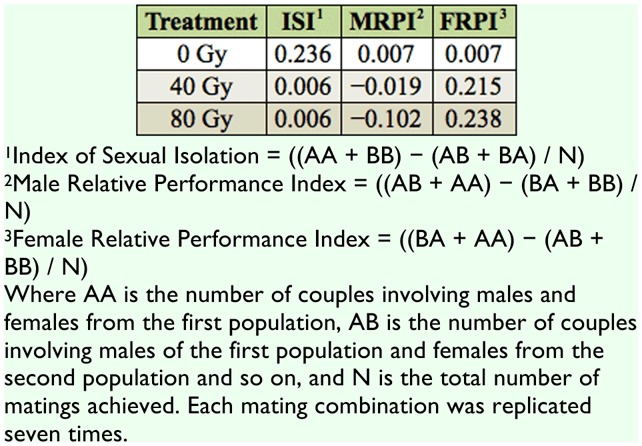
Sexual isolation indices (ISI, MRPI, and FRPI) for non-irradiated mass reared (0 Gy) and irradiated mass reared (40 and 80 Gy) *Anastrepha obliqua* released in field cages with wild males and females.

**Table 2.**
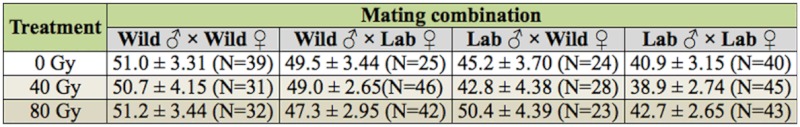
Duration ± SE in minutes of copulations according to mating combination (Wild ♂ × Wild ♀; Wild ♂ × Lab ♀; Lab ♂ × Wild ♀; Lab ♂ × Lab ♀) and radiation treatment (0, 40, or 80 Gy) for *Anastrepha obliqua* released in field cages.

**Figure 1.**
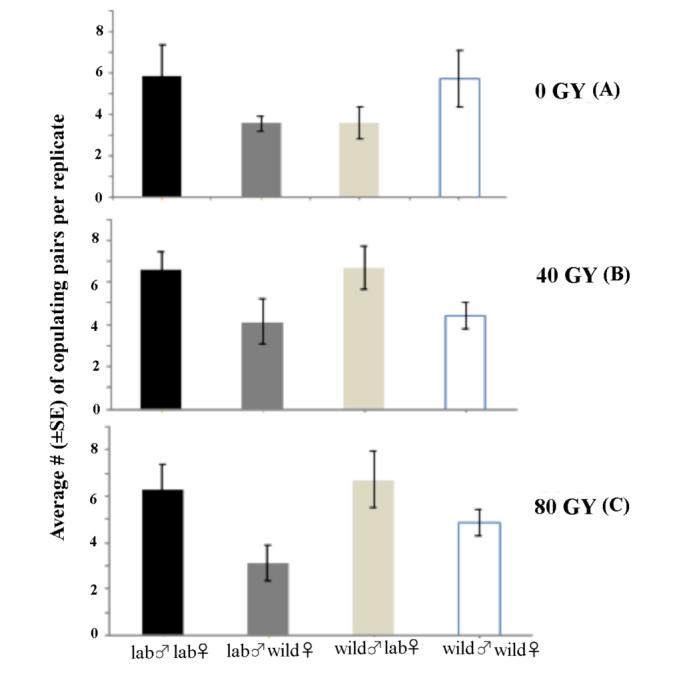
Average number ± SE of copulations per replicate according to mating combination (Wild ♂ × Wild ♀; Wild ♂ × Lab ♀; Lab ♂ × Wild ♀; Lab ♂ × Lab ♀) for cohorts of 30 male and female *Anastrepha obliqua* irradiated at (A) 0, (B) 40, or (C) 80 Gy. High quality figures are available online.

**Mating duration**. A factorial ANOVA revealed a significant effect of mating combination on duration of copulations (*F*_3,406_ = 5.62, *p* < 0.01), and no significant effect of treatment or the interaction between mating combination and treatment (*F*_2,406_ = 0.57, *p* = 0.562), (*F*_6,406_ = 0.36; *p* = 0.903) respectively. Irrespective of treatment, copulations were shorter when both laboratory and wild females mated with laboratory males than when they mated with wild males (Table 2).

**Figure 2.**
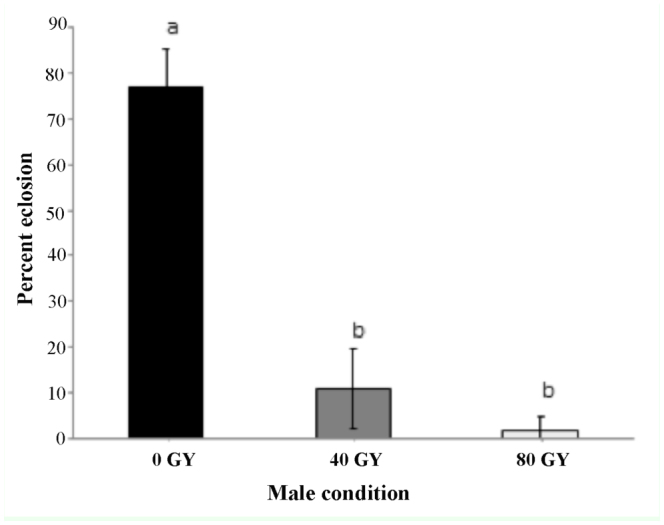
Average percent ± SE egg hatch of eggs laid by non-irradiated laboratory females crossed with laboratory males irradiated either at 0 (black bars), 40 (grey bars), or 80 Gy (white bars). Bars crowned by different letters represent significant differences at the 0.05 level. High quality figures are available online.

**Male sterility**. A repeated measures ANOVA revealed significant differences in fertility (% hatch) of non-irradiated females mated with males treated with different radiation doses (*F*_12,8_ = 74.722, *p* < 0.01). Females mated with non-irradiated males exhibited significantly greater fertility than those mated with males irradiated at 40 and 80 Gy, between which there were no differences in fertility (Figure 2).

## Discussion

In summary, our experiment revealed a strain effect on male *A. obliqua* mating performance. Laboratory males tended to mate less frequently with wild females than wild males. Duration of copulations was shorter when females of both strains (wild and laboratory) mated with laboratory males than when they mated with wild males, irrespective of radiation dose. Finally, although there were no statistical differences in fertility between females mated with males irradiated at low (40 Gy) or high (80 Gy) doses, percentage of egg hatch was unexpectedly high, probably reflecting a lack of uniformity in pupal age that could have obscured the potential effects of radiation dose on male performance.

Toledo et al. (2004) found that untreated laboratory females of *A. obliqua* crossed with males irradiated at 40 Gy 48 hours before emergence only produced 0.5% egg hatch, and those crossed with males irradiated at 75 Gy or more were completely non-fertile. Here, even for males irradiated at 80 Gy, 1.6% of eggs laid by untreated females hatched. The most likely explanation for this result is that some of the males irradiated in the tested mass rearing lots were in a much more advanced state of physiological development than 48 hours before emergence. In fact, adult emergence spanned a long period of time (> six days), a result that reflects rearing problems and severely compromises the ability of irradiating pupae at a physiological age when their developing reproductive tissues are susceptible to radiation damage. Williamson et al. (1985) found increasing proportions of fertility recovery for Medfly females irradiated at a 150 Gy dose 48, 24, and three hours before, as well as three hours after emergence. Toledo et al. (2004) also found that *A. obliqua* irradiated 24 hours before emergence at 60 Gy exhibited some degree of residual fertility (0.6%), while those irradiated 48 hours before emergence at the same dose were fully sterile. This explains the need of mass rearing facilities to apply high doses (80 Gy) for this species. To overcome this problem, measures have to be taken to homogenize pupal age, perhaps by reducing the length of the egg collection period by collecting larvae in water, or by subjecting pupae to longer low temperature periods after larval development during pupation.

High radiation doses negatively affect several species of fruit flies reared for SIT purposes (Lux et al. 2002; Rull et al. 2007; Collins et al. 2009; Dominak et al. 2010; Weldon et al. 2010). During a radiation dosimetry, Toledo et al. (2004) found that increasingly high radiation doses increasingly affected flight ability and survival of treated *A. obliqua*, a finding consistent with those of Collins et al. (2009) for *Bactrocera tryoni*. Weldon et al. (2010) found that both mass rearing history and irradiation resulted in an overall reduction of fly activity for *B. tryoni* and raised questions about how changes in activity levels could influence performance of mass-reared sterile flies in the field.

Here, an increase in copulations between irradiated females and wild males was observed in comparison to those between un-irradiated laboratory females and wild males. Unreceptive females in the genus *Anastrepha* have been observed to resist mating by vigorously shaking males after mounting before penetration (Rull et al. unpublished observations). Apparently, irradiated females were less able to reject courting males and engaged in more copulations than untreated females. Although this finding may not have intuitive implications for SIT success, it may have caused an experimental bias during evaluation of male performance. Although our design is well-suited to test sexual isolation between strains or populations, releasing laboratory and wild males competing for wild females only (no laboratory females) may be more informative on relative male performance. Ideally, when assessing mass reared strain performance, both designs should be used.

Despite the fact that our results might have been obscured by problems encountered during mass rearing that prevented irradiation at a proper age (48 hours before emergence), they nevertheless add to a now growing number of studies (Lux et al. 2002; Toledo et al. 2004; Rull et al. 2007; Collins et al. 2009) indicating that a reduction of radiation dose will result in greater male performance and greater sterility induction in wild populations. For this purpose, considering the fact that female *A. obliqua* are more radio sensitive than males, a minimum dose can be set higher than the dose at which egg production or hatch stops (Parker and Metha 2007).

While studying factors influencing the duration of refractory periods, Aluja et al. (2009) found that duration of copulations for *A. obliqua* was most influenced by male and female diet, and that females tended to mate for longer periods of time with males fed a poor diet than with those fed a rich diet. Here, males and females of both strains had *ad libitum* access to a rich diet, and yet females of both strains mated for shorter periods of time with laboratory males. Therefore, our results reflect clear differences between laboratory and wild males that are probably associated with artificial selection during mass rearing. Under the extremely crowded conditions and the reduced time window for reproduction encountered in mass rearing colonies, laboratory males engaging in shorter copulations have greater opportunity to inseminate more females and therefore have a selective advantage. Although for *A. obliqua* there is no association between sperm storage and copulation duration (Pérez-Staples and Aluja 2006), shorter copulations of laboratory males with wild females under an SIT scenario may affect transfer of other substances in the ejaculate (e.g., accessory gland products) that could affect the duration of the refractory period and therefore the efficiency of SIT.

A number of measures can be applied to address the effect of rearing history on male mating performance. Replacing strains to restore male mating competitiveness by forcing wild individuals into mass rearing results in a succession of bottlenecks that affect the genetic composition of the new strain (Parker 2005). In general, managers are reluctant to replace strains adapted to mass-rearing conditions, fearing reduced production from a newly colonized, non-laboratory-adapted strain. These problems can be overcome by using refreshment schemes, where laboratory females are crossed with wild males to produce a strain with renewed male mating competitiveness and yet retaining mass rearing adaptation of females (Rull and Barreda-Landa 2007).

To improve performance of *A. obliqua* males reared for SIT programs in Mexico, efforts have to concentrate on homogenizing pupal age, reducing radiation dose, and refreshing the mass rearing strain. Additionally, it would be interesting to compare different field cage methodologies to test sterile male mating performance. Ideally, when sufficient wild material is at hand, comparisons to test for strain isolation can be made by releasing both sexes of laboratory and wild strains, while releases of wild and laboratory males only, competing for copulations with wild females, can be used to assess relative male mating performance.

## Abbreviations

SIT: sterile insect technique
